# Hypoxia Induces a Prothrombotic State Independently of the Physical Activity

**DOI:** 10.1371/journal.pone.0141797

**Published:** 2015-10-30

**Authors:** Marisa Ninivaggi, Marieke de Laat, Marcus M. D. Lancé, Cécile H. Kicken, Leonie Pelkmans, Saartje Bloemen, Marlou L. Dirks, Luc J. C. van Loon, José W. P. Govers-Riemslag, Theo Lindhout, Joke Konings, Bas de Laat

**Affiliations:** 1 Department of Synapse bv, Maastricht University Medical Center, Maastricht, The Netherlands; 2 Department of Biochemistry, Maastricht University Medical Center, Maastricht, The Netherlands; 3 Department of Anesthesiology, Maastricht University Medical Center, Maastricht, The Netherlands; 4 Department of NUTRIM School of Nutrition and Translational Research in Metabolism, Maastricht University Medical Center, Maastricht, The Netherlands; Ottawa Hospital Research Institute, CANADA

## Abstract

Hypoxia (oxygen deprivation) is known to be associated with deep vein thrombosis and venous thromboembolism. We attempted to get a better comprehension of its mechanism by going to high altitude, thereby including the potential contributing role of physical activity. Two groups of 15 healthy individuals were exposed to hypoxia by going to an altitude of 3900 meters, either by climbing actively (active group) or transported passively by cable car (passive group). Both groups were tested for plasma fibrinogen, von Willebrand factor and factor VIII levels, fibrinolysis, thrombin generating capacity, heart rate, oxygen saturation levels and blood pressure. As a control for the passive group, 7 healthy volunteers stayed immobile in bed for 7 days at normoxic conditions. The heart rate increased and oxygen saturation levels decreased with increasing altitude. Fibrinolysis and fibrinogen levels were not affected. Factor VIII and von Willebrand factor levels levels increased significantly in the active group, but not in the passive group. Plasma thrombin generation remained unchanged in both the active and passive group with increasing altitude and during 7 days of immobility in healthy subjects. However, by applying whole blood thrombin generation, we found an increased peak height and endogenous thrombin potential, and a decreased lagtime and time-to-peak with increasing levels of hypoxia in both groups. In conclusion, by applying whole blood thrombin generation we demonstrated that hypoxia causes a prothrombotic state. As thrombin generation in plasma did not increase, our results suggest that the cellular part of the blood is involved in the prothrombotic phenotype induced by hypoxia.

## Introduction

Hypoxia is an imbalance between oxygen supply and consumption, which affects cellular viability and can lead to cellular dysfunction, cell death and (multiple) organ failure [1–3]. Hypoxia is known to be associated with a prothrombotic phenotype, especially in air travelers and mountaineers [4, 5]. However, oxygen deprivation also seems to play a role in mediating hypercoagulability in several pathologies, e.g. chronic obstructive pulmonary disease (COPD) and obstructive sleep apnea syndrome (OSAS) [6–10]. The increased risk for developing thrombosis could result from hypoxia-induced platelet aggregation and activation of blood coagulation [11, 12]. Fragmentation of a venous thrombus might cause pulmonary embolism or stroke, which is often the case in COPD and OSAS patients [2, 6–10].

In order to study the influence of hypoxia on haemostasis, several studies have been performed in the past with healthy individuals being exposed to lower oxygen pressure either by going to high altitude or by inducing hypobaric hypoxia. It is well known that hypobaric hypoxia due to air travel leads to the development of venous thrombosis [13]. The odd’s ratio varies between 2- and 6-fold depending of the seat location on the plane, obesity, duration and number of flights, gender, age, length, use of oral contraceptives, sleeping and coagulation defects (e.g. Factor V Leiden, prothrombin mutation, high factor VIII and IX levels, etc.) [12, 14]. However, the most relevant element that causes the absolute risk for the development of venous thrombosis after long-haul flight is still unknown [12]. In contrast to these results, there are also studies that showed an association between hypoxia and a reduced coagulation or even that hypoxia does not have any effect at all [15–18].

In previous studies two conceptual errors were made that might explain the conflicting data. First, at high altitude the change in barometric pressure has an effect on blood drawing when using the vacutainer system, as less blood will enter the tube, while the amount of anticoagulant remains constant [19]. In other words, the ratio blood:anticoagulant is no longer correct and thereby induces an artificial anticoagulant effect. Secondly, exercise, e.g. due to climbing or walking to a higher altitude induces an increase in von Willebrand factor and factor VIII (FVIII), causing an exercise-increased haemostatic response leading to a prothrombotic phenotype [20–23].

Our aim was to investigate the effect of hypoxia on haemostasis avoiding these two conceptual errors. We formed two groups: one that climbed to high altitude (3900 meters) and another that was transported to high altitude. In addition, blood coagulation was studied by assaying the thrombin generating capacity in plasma and whole blood, rather than performing clotting-time based tests that are less sensitive to changes in the blood clotting status of a subject [24]. As a control group, we also tested a group of healthy, young individuals that remained immobile in bed for 7 days at normoxic conditions. Understanding the mechanisms of hypoxia could lead to a better and more specific prophylactic treatment and hopefully to a prolongation of the life expectancy of many patients with pathologies in which hypoxia plays a major role. This is not only the case for patients with e.g. COPD and OSAS, who have an increased risk of dying of venous thromboembolism (VTE), but also for mountaineers and air travellers.

## Materials and Methods

### Study protocol

We performed a randomized controlled study to investigate the effect of hypoxia on coagulation. Healthy volunteers that participated in our study passed a physical check-up including an evaluation of medical history, blood pressure, oxygen saturation, cardiac function by an electrocardiogram and overall fitness by an exercise test. Exclusion criteria were: medication interfering with coagulation, a history of cardiovascular disease, having mobility impairment (being able to walk without aid or without discomfort), being younger than 18 years or older than 50 years and not meeting the demands of physical check-up. Healthy volunteers with climbing experience (N = 30) were included and randomized into two groups after age and gender stratification. The first group was called the “active” climbing group that ascended by actively climbing (group A). The second group was called the “passive” group that increased in altitude by cable car (group B). This approach was chosen in order to investigate the role of exercise in the process of altitude-induced hypoxia, as a cause of a hypercoagulable state. Blood samples were taken at different heights: at 50 meters, 1100 meters, 2045 meters, 3100 meters and 3900 meters. Blood drawing and testing was done on the same day for both groups to exclude day to day variability. Blood pressure, heart rate and blood oxygen saturation levels were recorded by trained medical personnel on each testing day. The volunteers had to complete a questionnaire on every testing day concerning their physical and mental condition (according to the Lake Louise Consensus on the Definition of Altitude Illness). In the total study duration of 8 days, 2 participants (one of each group) had to leave the study at 3100 meters above sea level as they suffered from acute mountain sickness. The study was approved by the Medical Ethical Committee of the Maastricht University Medical Centre and by the local authorities (METC number NL43244.068.13). The study met all institutional ethics requirements according to the Helsinki declaration (2008).

### Resting control group

To investigate the effect of passive behaviour on hemostatic values we drew blood samples from 7 healthy adults that remained immobile in bed for 7 days at normoxic conditions. During the entire bed-rest period, volunteers were allowed to sit semi-supine or supine upon their preference and perform non-weight bearing movement within the bed. However, subjects were not allowed to get out of the bed or to perform any weight bearing exercise for the entirety of the bed-rest period. Blood samples were taken on day 1, day 3 or 5, day 7 and day 8 (right before the end of the study). This part of the study was also approved by the Medical Ethical Committee of the Maastricht University Medical Centre and by the local authorities (METC number NL48569.068.14).

### Blood collection

Blood was drawn after obtaining written informed consent. Blood (9 volumes) was aseptically drawn using vacutainer tubes (Greiner Bio-One) containing 3.2% sodium citrate (1 volume), from the antecubital vein of healthy subjects. The blood was kept at room temperature (± 22°C) and used within 2 hours. Platelet poor plasma was obtained by centrifugating twice at 2630 × g for 10 min. Plasma samples were stored at -80°C.

### Coagulation factor levels and fibrinolysis assessment

Von Willebrand factor (antigen) and Factor VIII (FVIII activity) were measured in plasma with the STA-R evolution (Diagnostica Stago). Plasma fibrinogen levels were measured by the Clauss method [25]. Fibrinolysis was measured as follows: plasma samples (75 μl) were transferred into low binding polystyrene 96-well plates (Greiner, Frickenhauser, Germany) and pre-heated for 10 min at 37°C. Fibrin polymerization and fibrinolysis was started by addition of 75 μl activation mixture containing 1 nM thrombin (Enzyme Research Laboratories), 10 μM phospholipid vesicles (1,2-dioleoyl-sn-glycero-3-phosphoserine (DOPS), 1,2-dioeoyl-sn-glycero-3-phosphocholine (DOPC), and 1,2-dioleoyl-sn-glycero-3-phosphoethanolamine (DOPE) (DOPS/DOPC/DOPE, 20 mol/60 mol/20 mol), 100 ng/ml tPA and 16 mM CaCl_2_ (final concentrations). Turbidity was measured at 405 nm every 15 sec for 180 min at 37°C using an ELx808 plate reader (Biotek Instruments, Winooski, VT). The clot lysis time (CLT) was calculated as the time from 50% clot formation to 50% fibrinolysis. Samples were measured in triplicate.

### Thrombin generation in plasma

Thrombin generation in plasma was measured with the Calibrated Automated Thrombogram assay as previously described [23]. Briefly, 80 μl of platelet poor plasma was mixed with 20 μl of a mixture containing 6 pM tissue factor (TF, Dade-Behring) and 24 μM phospholipid vesicles (DOPS/DOPC/DOPE; 20/60/20 mol%/mol%/mol%; Avanti). After 5 minutes of incubation at 37°C, thrombin generation was started with 20 μl of the activator containing 100 mM CaCl_2_ and 2.5 mM of the thrombin specific substrate, Z-Gly-Gly-Arg-7-amino-4-methylcoumarin (Bachem). Fluorescence was measured with a Fluoroscan Ascent reader (Thermo Labsystems). Samples were run in triplicate and each curve was calibrated to correct for inner-filter effects and substrate consumption. All procedures were performed at 37°C and data were analyzed with dedicated software (Thrombinoscope, Stago).

### Whole blood thrombin generation

Whole blood thrombin generation was performed as previously described [26]. Briefly, 30 μl of citrated whole blood was mixed with 10 μl thrombin specific substrate (P_2_Rho; 1.8 mM) and either with 20 μl TF and CaCl_2_ (1.5 pM and 50 mM, respectively) or with 20 μl human thrombin calibrator (α_2_M-T, 300 nM thrombin activity). Immediately after mixing, 5 μl of the sample was added to paper disks (Whatman GmbH), which were placed in an 96 well plate, and covered with 40 μl of mineral oil (USB Corporation) to prevent evaporation. Samples were analyzed for 50 minutes and fluorescence was recorded every 6 seconds with a Fluoroskan Ascent microplate fluorometer with λ_ex_ = 485 nm and λ_em_ = 538 nm (Thermolabsystems). Samples were tested in triplicate and calibrated as previously described [27]. All procedures were performed at 37°C and thrombin generation curves were calculated as previously described [28].

### Statistical analysis

The Wilcoxon matched-pairs signed rank test was used for significance testing. P-values <0.05 were considered to be statistically significant. Data given are medians interquartile ranges unless otherwise indicated.

## Results

### Effect of altitude on the amount of blood entering the vacutainer

The barometric pressure decreases when ascending (S1A Fig), and therefore the oxygen levels decrease (S1B Fig). A hypobaric environment induces difficulty in blood drawing as vacutainers depend on a difference in pressure between the inside of the tube and the outside. We found that the blood tubes were not completely filled at higher altitudes resulting in a slightly higher citrate concentration compared to baseline at 50 meters above sea level (S1C Fig). Therefore, we performed a control experiment in which we investigated whether we had to correct for these higher citrate concentrations in our assays. Blood taken from a healthy donor was supplemented *in vitro* with citrate up to the same concentration as at 50, 3100 and 3900 meters above sea level and we also used two higher concentrations that corresponded to 4400 meters and 4660 meters above sea level. Thrombin generation in plasma and whole blood were not affected by the increase of citrate at the altitudes on which blood samples were taken for this study (S2 Fig). This is probably due to the excessive amount of calcium used in our assay, which corrects for small differences in citrate concentrations in our samples. A small decrease in plasma thrombin generation was observed in the highest citrate concentration corresponding to approximately 4660 meters of altitude, indicating that from that point onward the plasma based thrombin generation assay becomes sensitive to the increasing citrate concentrations.

### Effect of altitude induced hypoxia on oxygen saturation levels, heart rate and blood pressure

**Fig 1:**
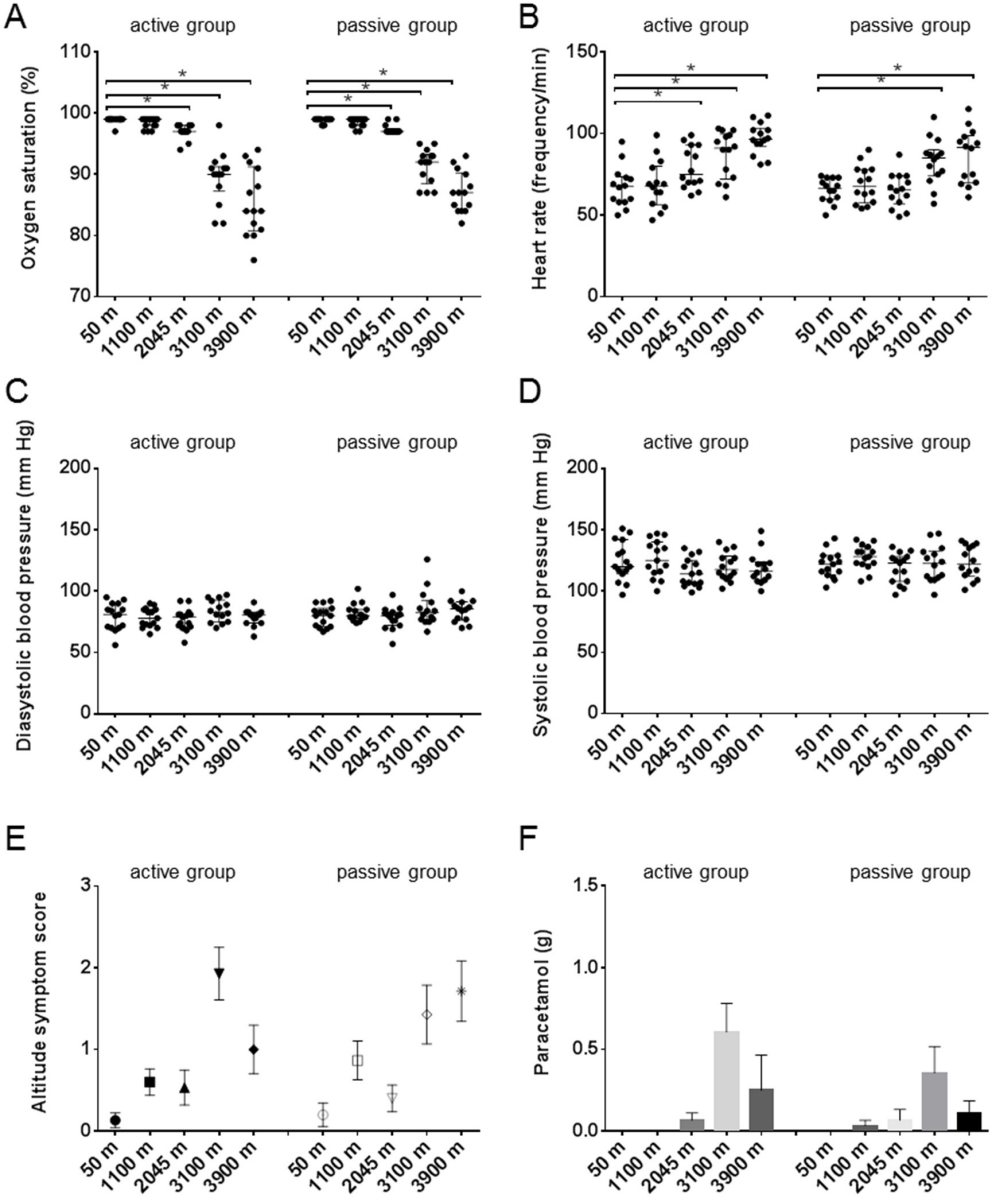
Effect of hypoxia on oxygen saturation, heart rate, blood pressure, altitude symptom score and paracetamol intake. Data are medians with interquartile ranges (A-D) or mean with SEM (E-F). *p<0.05.

There were no differences between the active group and the passive group in baseline characteristics (Table 1). Measurement of the oxygen saturation levels in capillary blood revealed that both groups suffered from hypoxia to the same extent starting at 2045 meters of altitude, medians (interquartile ranges) were 97% (97–98) for the active group and 97% (97–97) for the passive group (Fig 1A, p <0.0001 for both groups compared to baseline at 50 meters 99% (99–99)). The oxygen saturation levels decreased down to 84% (81–91) for the active group and 87% (84–90) for the passive group at 3900 meters. Adaptation of the body to hypoxia by increasing the heart rate started at 2045 meters for the active group (77/min (70–93), p = 0.0129) in contrast to the passive group that showed an increased heart rate from 3100 meters of altitude onwards (85/min (74–90), p = 0.0004) compared to baseline values 67/min (59–74) (Fig 1B).

**Table 1:** Basic characteristics of both groups at 50 m (day 1). Data are mean values ± SD.

Baseline	Active group	Passive group
Gender (male/female)	7/8	7/8
Age (years)	31.7 ± 5.7	28.0 ± 5.2
Hematocrit (%)	41.3 ± 5.1	41.6 ± 3.3
Hemoglobin (g/L)	8.7 ± 1.1	8.8 ± 0.7
Heart rate (min^-1^)	69 ± 13.6	67 ± 10.9

Systolic and diastolic blood pressure were not affected by altitude (Fig 1C and 1D). To detect early signs of altitude sickness, the participants completed a questionnaire on each testing day at every altitude (based on the Lake Louise Consensus on the Definition of Altitude Illness). We found an increase in symptoms related to altitude sickness with increasing altitude up to 3900 meters (Fig 1E). In addition, intake of paracetamol showed the same trend up to 3100 meters (Fig 1F). The lower intake of paracetamol at 3900 meters might be related to the fact that the testing day at 3900 meters was the last day of the study and participants knew they would descend the same day.

### Coagulation factor levels and fibrinolysis in plasma

**Fig 2:**
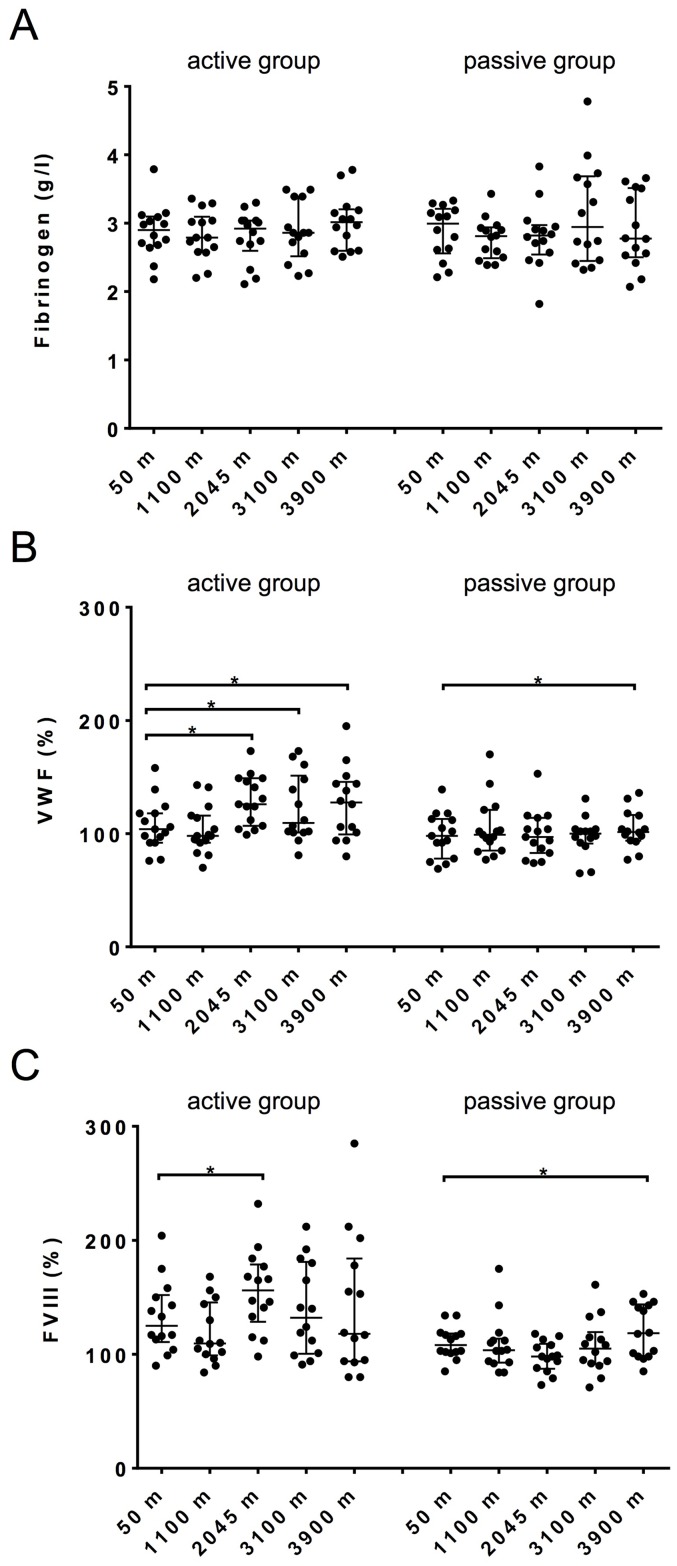
Fibrinogen (A), von Willebrand factor (B) and FVIII (C) levels in plasma. Data are medians with interquartile ranges. *p<0.05.

Fibrinogen levels were unaffected by hypoxia in both groups on each testing day. The median fibrinogen level was 3 g/l (2.7–3.1) for the active group and 2.9 g/l (2.4–3.2) for the passive group at baseline. At 3900 m the fibrinogen values were respectively 3 g/l (.6–3.2) and 2.8 g/l (2.5–3.5) (Fig 2A). We found that both von Willebrand factor levels and FVIII gradually increased in the active group starting from 2045 meters (Fig 2B and 2C; von Willebrand factor: 126% (107–149) compared to baseline levels 104% (92–118); and FVIII: 156% (129–179) compared to baseline levels 125% (111–152)). At 3100 and 3900 meters FVIII levels were not significantly different compared to baseline values. Fibrinolysis was determined as the CLT. Since the CLT was not altered in both groups with increasing hypoxia, this would indicate that hypoxia does not affect fibrinolysis (S3 Fig).

### Thrombin generation

**Fig 3:**
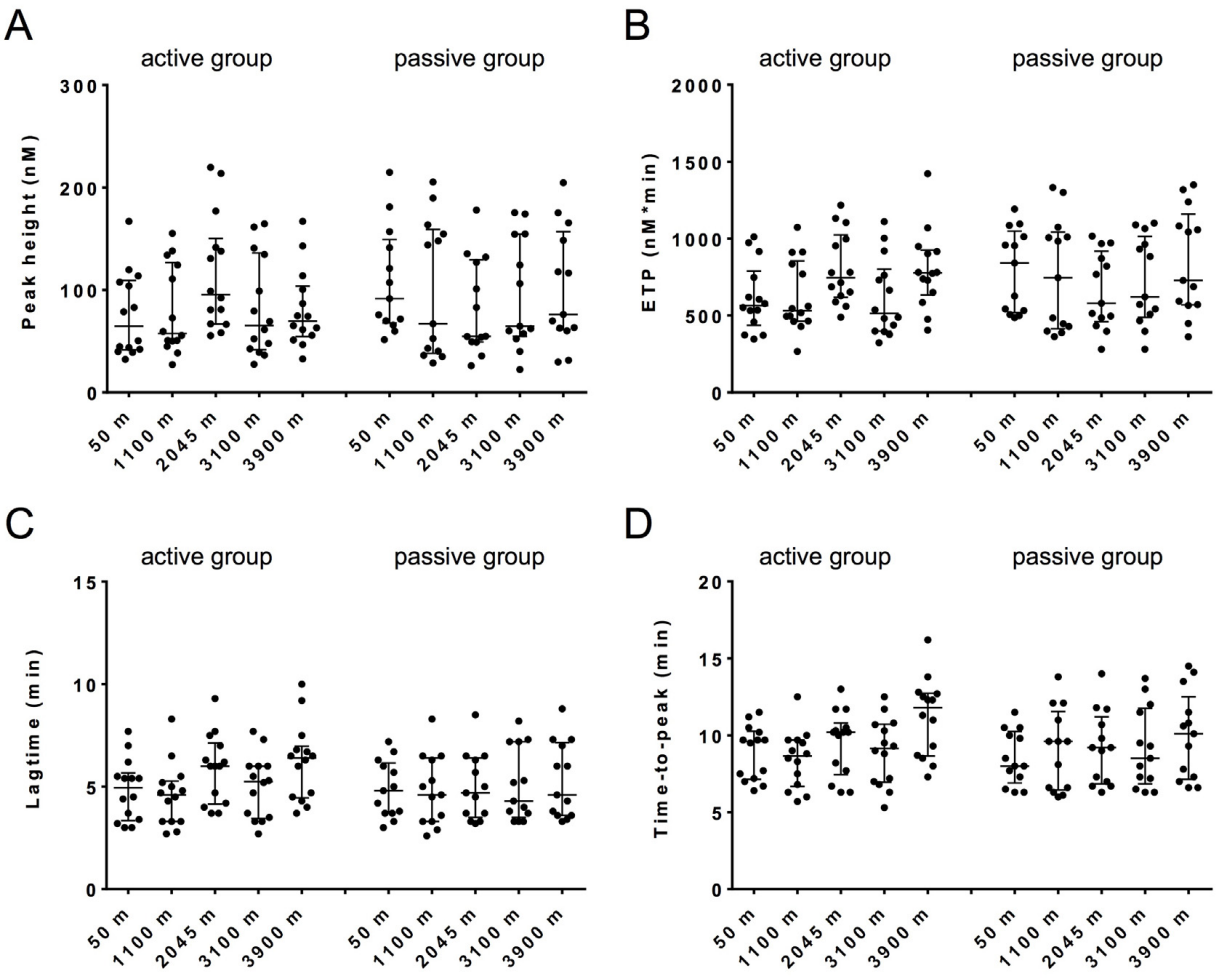
Thrombin generation in plasma. Thrombin generation was started with 1 pM TF, 4 μM phospholipid vesicles and 16.7 mM CaCl_2_. The parameters depicted are the Peak height (A), ETP (B), Lagtime (C) and Time-to-peak (D). Data are medians with interquartile ranges.

To investigate a possible association between hypoxia and blood coagulation we applied thrombin generation in platelet poor plasma and whole blood. For both the active and passive group we found a high variation at baseline between individuals that is comparable with other studies [26, 29]. Thrombin generation measured in platelet poor plasma revealed no relevant differences in both groups for all parameters at different altitudes (Fig 3).

**Fig 4:**
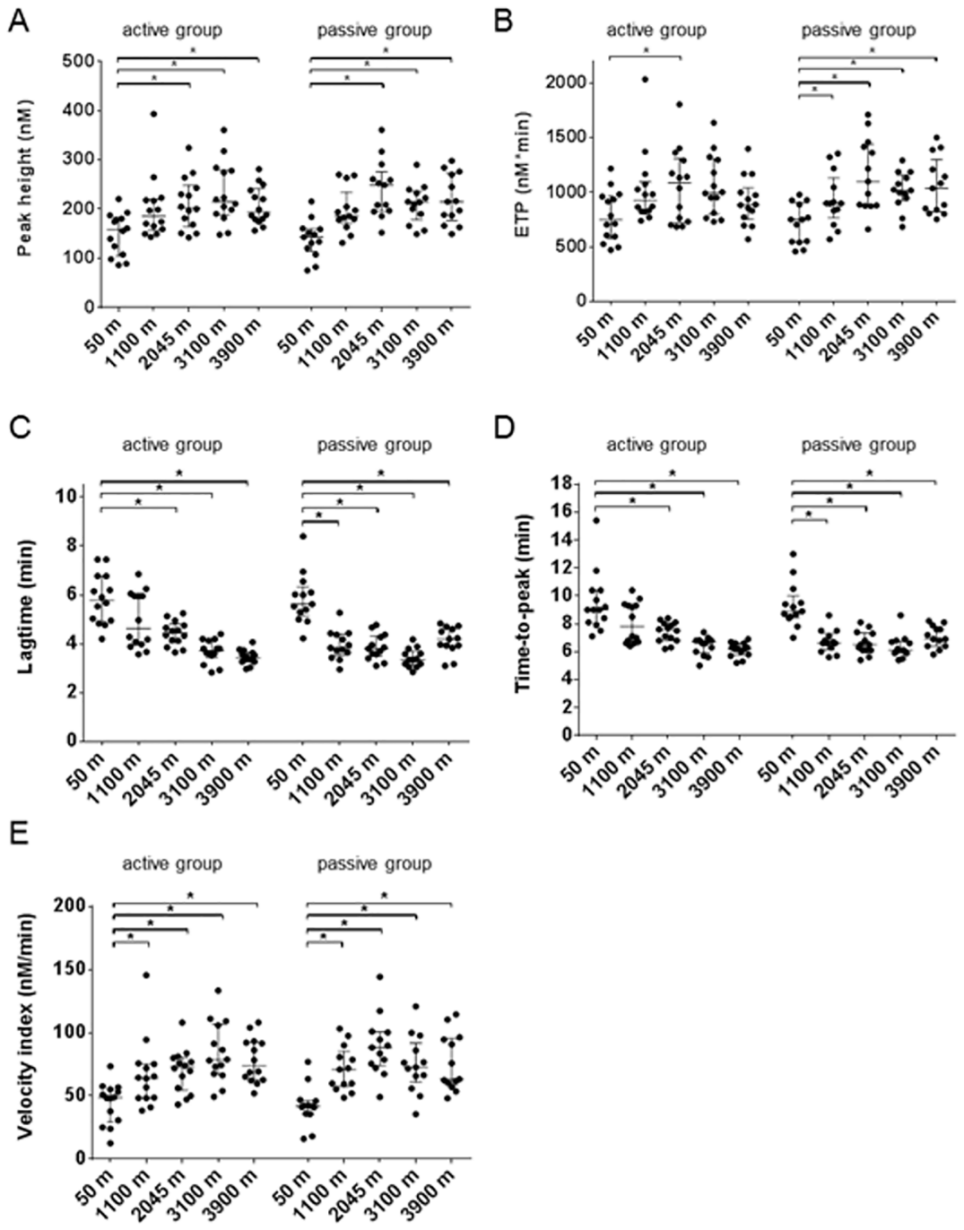
Thrombin generation in whole blood. Thrombin generation was started with 0.5 pM TF and 16.7 mM CaCl_2_. The parameters depicted are the Peak height (A), ETP (B), Lagtime (C), Time-to-peak (D) and Velocity index (E). Data are medians with interquartile ranges.

Recently we developed a method to measure thrombin generation in whole blood, which has a major advantage as it also takes the cellular portion of the blood into account [26]. In contrast to the results seen in plasma, thrombin generation in whole blood revealed significant differences in thrombin generation parameters for both groups with increasing altitudes. We observed a gradual increase in peak height while ascending for both the active group (baseline 158 nM (105–182) compared to 3900 meters 194 nM (178–241)) and the passive group (baseline 143 nM (114–160) compared to 3900 meters 215 nM (177–273)), reaching a plateau at 2045 meters (Fig 4A). As for the peak height, the ETP also showed a gradual increase with increasing altitude in both groups (baseline 747 nM*min (578–965) compared to 3900 meters 884 nM*min (752–1039)) and the passive group (baseline 749 nM*min (544–906) compared to 3900 meters 1035 nM*min (822–1298)), reaching a plateau at 2045 meters (Fig 4B). The lagtime and time-to-peak were shortened with increasing altitude. The lagtime of the active group decreased from 5.8 min (4.92–6.77) at baseline down to 3.4 min (3.27–3.66) at 3900 meters and for the passive group from 5.6 min (5.15–6.32) down to 4.2 min (3.88–4.65) at 3900 meters (Fig 4C). The time-to-peak of the active group decreased from 9.0 min (8.1–10.4) at baseline down to 6.2 min (5.8–6.6) at 3900 meters and for the passive group from 8.9 min (8.5–10) down to 6.9 min (6.4–7.8) at 3900 meters (Fig 4D). The velocity index, which is a measure for the velocity of thrombin formation, increased from 49 nM/min (29–55) at baseline up to 73.8 nM/min (63–92) at 3900 meters for the active group and from 42 nM/min (36–46) up to 63 nM/min (59–96) at 3900 meters for the passive group (Fig 4E). Since the prothrombotic phenotype was seen on both the active and passive group this would indicate that this effect was predominantly due to hypoxia and not to exercise. Although all parameters were significantly different from baseline at the highest altitude (3900 meters), the influence of hypoxia on these parameters seems to achieve a maximal effect already at 2045–3100 meters.

Statistical analysis of the data between the active and passive group revealed no significant differences at baseline for each thrombin generation parameter (p-values ranged from 0.26 to 0.41). At 3900 meters, ETP and peak height values were not significantly different between the two groups (p-value were 0.4 and 0.41, respectively), but lagtime and time-to-peak were longer for the passive group (p <0.05).

### Resting control group

**Fig 5:**
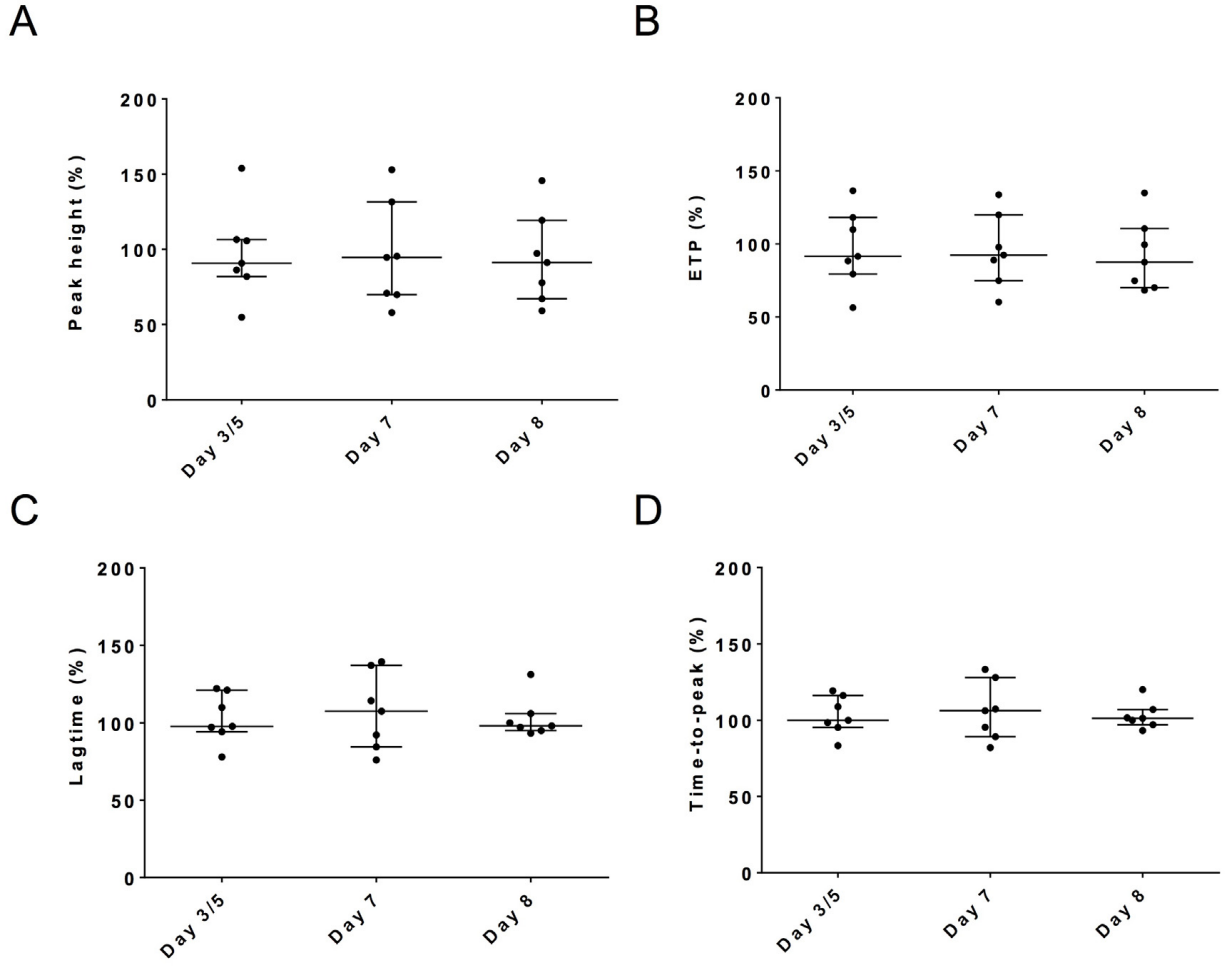
Whole blood thrombin generation parameters of the resting control group. Thrombin generation was started with 0.5 pM TF and 16.7 mM CaCl_2_. The parameters depicted are the Peak height (A), ETP (B), Lagtime (C) and Time-to-peak (D). Data are median percentages normalized to day 1 with interquartile ranges (N = 7).

The resting control group consisted of 7 healthy donors with a mean age of 23 (± 2 SD). They remained immobile in bed for 7 days at normoxic conditions. Thrombin generation was analysed in whole blood, however no differences could be observed between the different measuring days for every thrombin generation parameter (Fig 5).

## Discussion

Hypoxia has been indicated to be associated with venous thromboembolism [11, 30]. In our study, thrombin generation in whole blood indicated a prothrombotic phenotype (increased peak height and ETP, decreased lagtime and time to peak) with increasing hypoxic conditions. We did not detect this prothrombotic state measuring TG in plasma indicating that the cellular portion of the blood is responsible for the increased thrombotic risk.

Several studies have been performed to investigate the effect of hypoxia on the human body. However, contrasting results were found in these studies, but most found a prothrombotic phenotype [15–18]. Many studies included only participants that reached certain altitudes actively and it is known that physical activity induces a prothrombotic phenotype. We decided to correct for this potential confounder by dividing our subjects into two groups, an active and a passive ascending group, in order to discriminate the effects caused by pure hypoxic conditions or by a combination of hypoxic conditions plus exercise. Plasma FVIII and von Willebrand factor concentrations only increased with altitude in the active group which supports this assumption. This was also seen by statistical analysis of the whole blood thrombin generation parameters between the two groups. At each height, no significant changes could be observed in ETP and peak height between the active and passive group. Lagtime and time-to-peak were significantly shorter in the active group at 3900 meters compared to the passive group, probably due to an increase in FVIII. However, for both groups peak height, ETP and velocity index increased gradually with increasing altitude, while the lagtime and time-to-peak decreased. As this observation was made in both groups, the elevated functional thrombin generation parameters were a result of hypobaric hypoxia and did not relate to exercise. Comparably, the results of our resting control group revealed no differences in the whole blood thrombin generation parameters, which is in line with literature [31–34]. Schreijer et al., showed that coagulation was activated in 17% of the air travelers compared to 3% of a group of healthy volunteers after an 8-hour period of prolonged sitting [14, 35]. These findings are in contrast to what was generally believed, namely that immobilization causes a shift to a more prothrombotic phenotype, with a high risk for developing deep vein thrombosis [36, 37]. Nonetheless, patients with a neurological immobility were recently found to have an increased risk of developing thrombosis [38].

Rosendaal et al.[5]. published that all long-duration travel, either by car, bus or airplane, is associated with an increased risk of venous thrombosis. Traveling by plane was associated with the highest risk (up to 6 times increased risk) indicating a role for hypobaric hypoxia [5, 12, 14, 35, 39]. In contrast, Toff et al.[40]. performed a study in which healthy volunteers were exposed to hypobaric hypoxia resembling the air pressure in the flight cabin. Interestingly, they did not find any relation between hypoxia and plasma markers such as thrombin-antithrombin complexes, von Willebrand factor, d-dimer and plasma thrombin generation. This is in line with our findings as the participants in our study had reduced oxygen saturation levels at 3100 meters, which corresponds to a lower barometric pressure than the cabin pressure during a flight (which usually resembles the pressure at 1500–2500 meters of altitude). In our study we induced hypoxia by increasing in altitude up to 3900 meters corresponding to 12.7% oxygen compared to 21% at sea level. Oxygen saturation in capillary blood started to decrease gradually from 2045 m to 3900 m, with the lowest levels at 84% (81–91) in the active group and 87% (84–90) in the passive group.

It was expected that under hypobaric conditions vacutainer tubes would not fill completely. Therefore we tested how much blood could be collected into the tubes at the different altitudes. On higher altitudes we found that indeed the blood tubes did not fill completely. Therefore, the increased sodium citrate levels already present in the tube could induce an artificial anticoagulant effect. We extrapolated that starting from approximately 4660 meters of altitude this phenomenon is able to influence haemostatic assays such as thrombin generation in plasma. As the citrate concentration corresponding to 3900 m of altitude did not affect our assays, there was no need for correction. Studies that exceed altitudes of 4660 meters should either correct for the citrate concentration or draw blood using a system that is not based on vacuum. We suggest this should be investigated for every functional assay.

An aspect that we did not investigate, but which we will examine in the future, is hypoxia/reoxygenation and its effect on haemostatic parameters. Recently Brill et al.[4]. performed a study in which mice were kept in a hypoxic environment (6% oxygen) for 24 hours followed by a reoxygenation period. They found that after induction of 1 hour stenosis to the inferior vena cava, thrombus size and prevalence was dramatically increased in these mice compared to control animals that were kept at normoxic conditions [4]. There are also several case reports of patients that suffered from venous thrombosis when returning from the mountains or after air travel. Whether these thrombi were formed during or after hypobaric pressure (either in plane or mountains) is not known. Interestingly, Rosendaal and coworkers found asymptomatic thrombi in passengers after air travel using ultrasound, indicating that these thrombi were formed during hypobaric situations [5].

One of the limitations of our study is probably that we did not take into account the intake of fluid and/or the urine production, as this can influence the blood rheology. However, we did encourage our participants to sufficiently hydrate, especially to prevent the development of acute mountain sickness. The hematocrit remained stable over time, indicating that dehydration was probably not present in any of our volunteers (data not shown). Another study limitation is the variability in physical condition of the persons in the active group. Some climbing parts were difficult for some persons and easy for others. This can also explain the variability seen in FVIII and von Willebrand factor in the active group. In conclusion, we have found clear proof that hypoxia is associated with an increased thrombin generation in whole blood, irrespective of exercise (active or passive). As we did not find a comparable increase in thrombin generation in the plasma, we have strong indications that the cellular part of blood is responsible for this prothrombotic phenomenon.

Further investigation is needed to elucidate which cells are involved and what their role is during hypoxic conditions on haemostasis. This will be studied more in depth with specific techniques, as well as the hypoxia/reoxygenation effect. Understanding the mechanisms of hypoxia could provide us with more information how to reduce or prevent hypoxia associated thrombotic risk. The importance is not limited to mountaineers and air travelers, but also includes pathologies in which hypoxia plays a major role (e.g. COPD and OSAS patients).

## Supporting Information

S1 Fig Barometric pressure effect at different altitudes on oxygen saturation levels and blood tube volumes. (A) Relation between barometric pressure and different altitude levels. (B) The oxygen saturation (%) for both groups measured at different barometric pressures. (C) The volume capacity of the blood collection tubes were measured on each altitude 10 times to investigate the effect of lower barometrical pressure on tube filling. Data are mean values with SD.
(TIF)

S2 Fig Thrombin generation measured in whole blood containing various amounts of citrate. Thrombin generation was measured in recalcified whole blood (A) and plasma (B) of samples containing varying citrate concentrations. Whole blood thrombin generation was measured with 0.5 pM TF and 16.7 mM CaCl_2_, while thrombin generation in plasma was activated with 1 pM TF, 4 μM phospholipid vesicles and 16.7 mM CaCl_2_. Samples were run in triplicate and the mean values of the thrombin generation curves are depicted.
(TIF)

S3 Fig Fibrinolysis in plasma samples. The CLT was measured as described. Data are medians with interquartile ranges.
(TIF)
