# Supplementary figures and images for: Hypoxia Induces a Prothrombotic State Independently of the Physical Activity
Source: PLoS One. 2015 Oct 30;10(10):e0141797. doi: 10.1371/journal.pone.0141797 (PMC4627841; doi:10.1371/journal.pone.0141797)

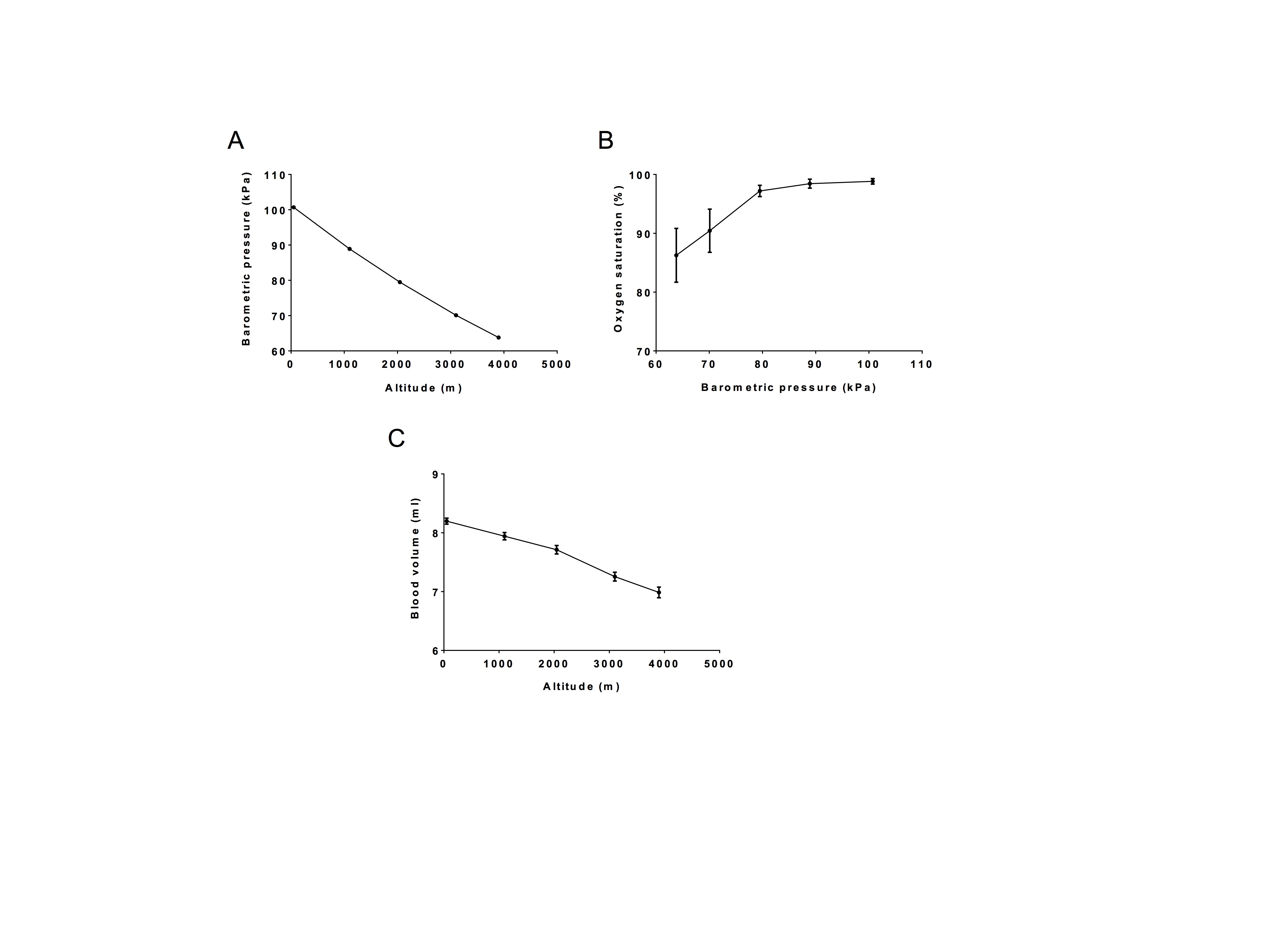

Supplement: S1 Fig — (A) Relation between barometric pressure and different altitude levels. (B) The oxygen saturation (%) for both groups measured at different barometric pressures. (C) The volume capacity of the blood collection tubes were measured on each altitude 10 times to investigate the effect of lower barometrical pressure on tube filling. Data are mean values with SD. (TIF) [file pone.0141797.s001.tif]

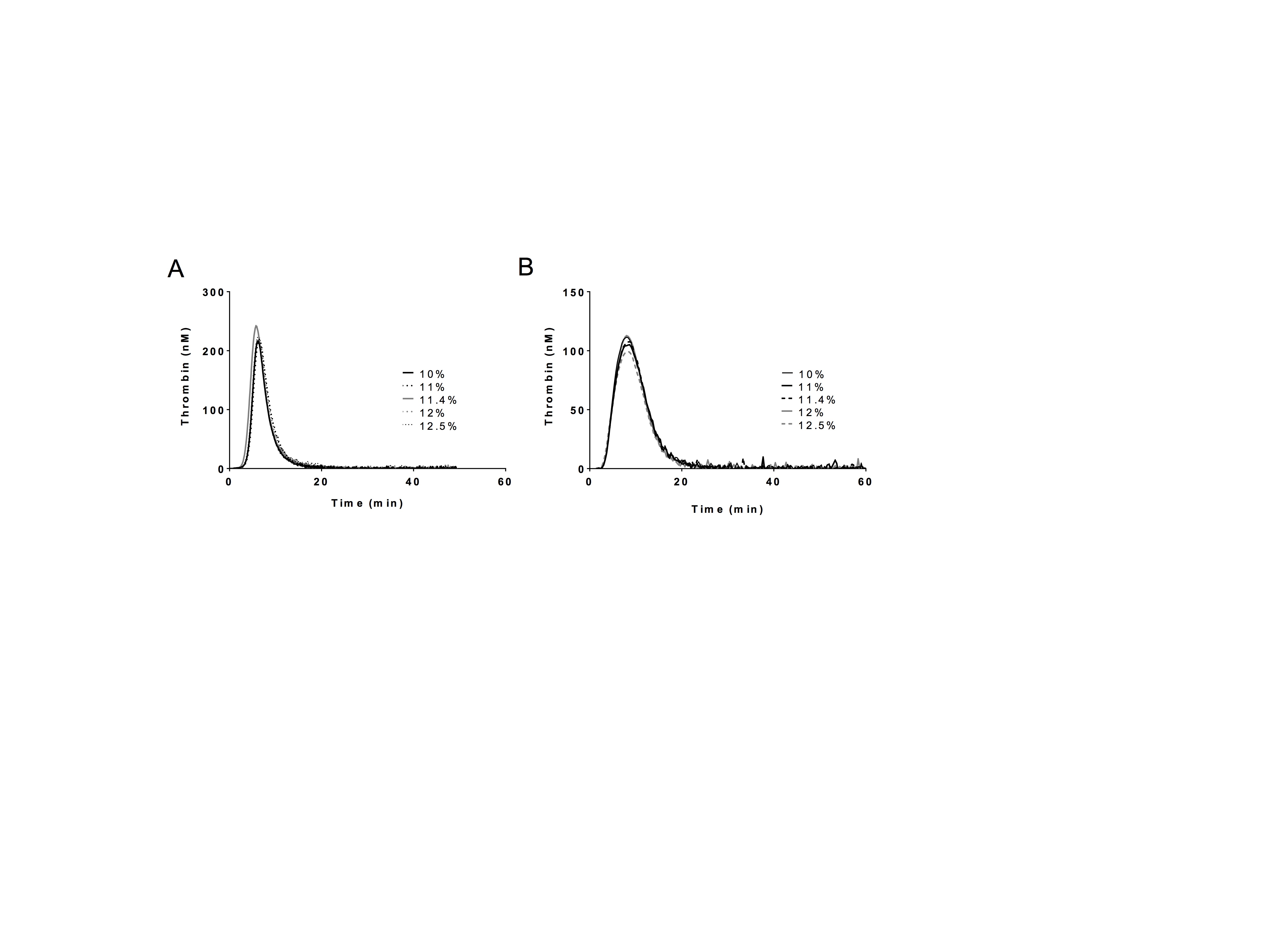

Supplement: S2 Fig — Thrombin generation was measured in recalcified whole blood (A) and plasma (B) of samples containing varying citrate concentrations. Whole blood thrombin generation was measured with 0.5 pM TF and 16.7 mM CaCl2, while thrombin generation in plasma was activated with 1 pM TF, 4 μM phospholipid vesicles and 16.7 mM CaCl2. Samples were run in triplicate and the mean values of the thrombin generation curves are depicted. (TIF) [file pone.0141797.s002.tif]

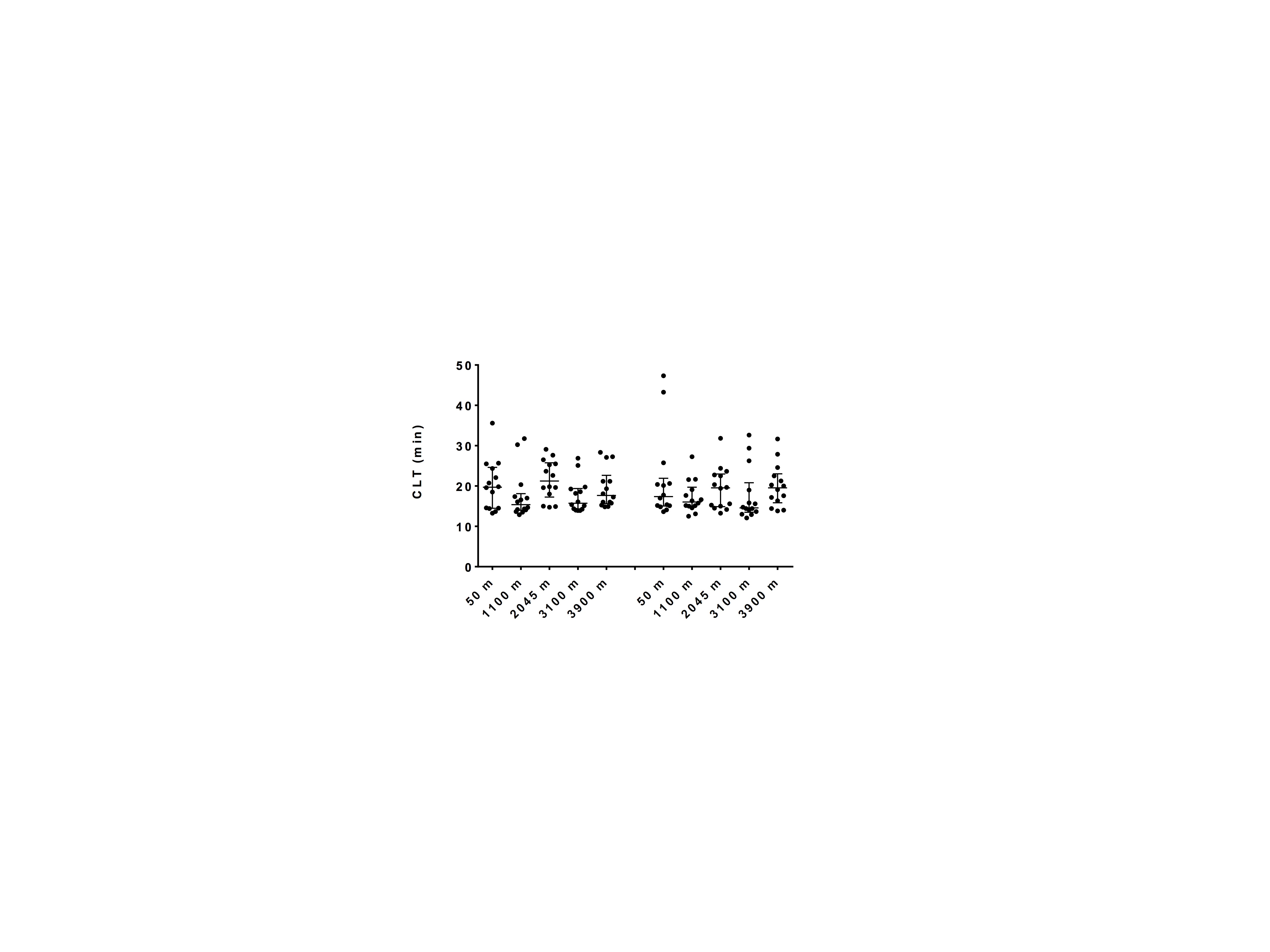

Supplement: S3 Fig — The CLT was measured as described. Data are medians with interquartile ranges. (TIF) [file pone.0141797.s003.tif]
